# Supplementary material for: Massively parallel digital transcriptional profiling of single cells
Source: Nat Commun. 2017 Jan 16;8:14049. doi: 10.1038/ncomms14049 (PMC5241818; doi:10.1038/ncomms14049)
Supplement: Supplementary Information — Supplementary Figures 1-10, Supplementary Tables 1-6 and Supplementary References [file ncomms14049-s1.pdf]

Supplementary Figures

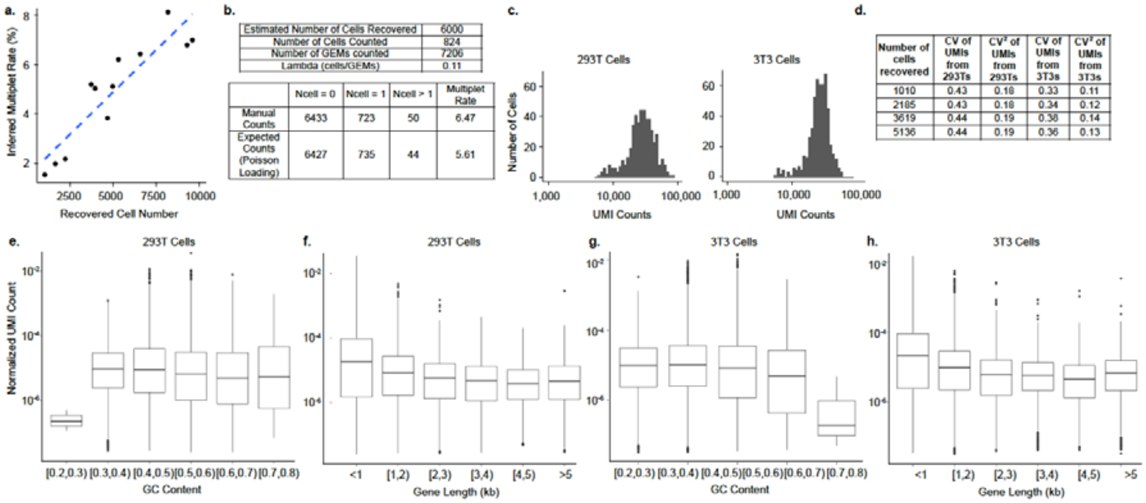

Supplementary Figure 1. Multiplet rate and sensitivity of the GemCode single cell platform from scRNA-seq of 50:50 mixing of 293Ts and 3T3s. (a) Inferred multiplet rate as a function of recovered cell number. **(b)** Expected (Poisson sampling) and observed (manual counting) number of cells per GEM. Ncell, number of cells in each GEM. **(c)** UMI count distribution of 293T cells (left), and 3T3 cells (right) in the 293T and 3T3 cell mixing sample. **(d)** CV and CV^2 of UMIs from 293Ts and 3T3s of 4 independent experiments. Distribution of normalized UMI counts vs. GC content **(e)** and gene length **(f)** in 293T cells. UMI counts were normalized by RNA content (Online Methods). Distribution of normalized UMI counts vs. GC content **(g)** and gene length **(h)** in 3T3 cells. Only genes with at least 1 UMI count detected in at least 1 cell are used. UMI normalization was performed by first dividing UMI counts by the total UMI counts in each cell, followed by multiplication with the median of the total UMI counts across cells. If there are multiple transcripts for a gene, the maximum length of the transcripts is used. Mean of GC content is calculated for each gene.

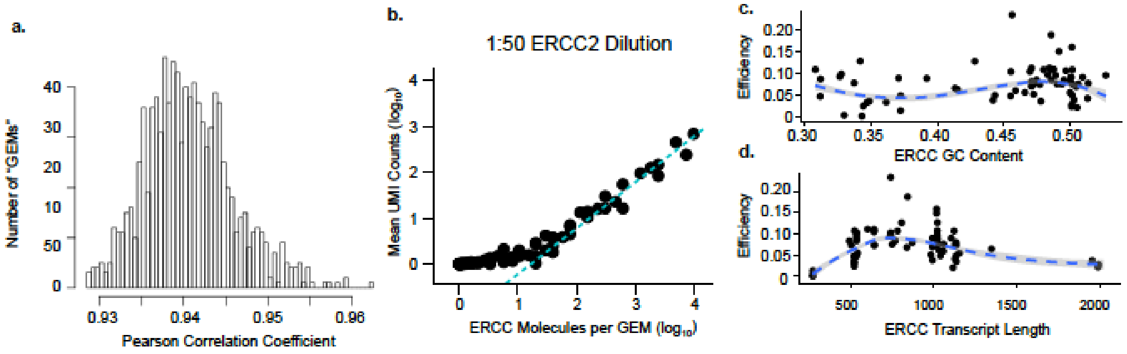

e.

Gene	GC (%)	Length	RNA Copies Inferred Based on Bulk RT ddPCR Data	cDNA Copies Calculated from GEM-RT ddPCR Data	Conversion Efficiency (Assuming 50% Efficient RT-ddPCR)
AP1S3	42.40	830	1.21-1.27	0.271-0.276	21.3-22.8
AP2MI	57.80	2037	11.83-14.76	1.60-1.65	10.8-14.0
DOLPP1	54.10	1931	8.98-9.81	0.93-0.98	9.4-10.9
ITSN2	39.90	6300	5.07-5.80	0.4-0.44	6.9-8.7
KPNA6	44.20	7378	6.60-6.94	0.48-0.54	6.9-8.1
LCMT1	45.40	1368	6.45-7.26	1.43-1.65	19.7-25.5
ORAOV1	39.80	3948	0.95-1.05	0.17-0.18	16.0-18.6
SERAC1	53.50	2478	0.99-1.13	0.03-0.05	2.5-5.1

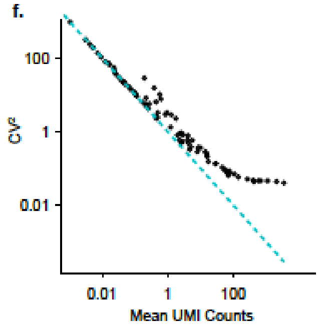

Supplementary Figure 2. Conversion efficiency of the GemCode single cell platform. (a) Distribution of Pearson correlation coefficient between expected vs. observed UMI counts for all GEMs, mean=0.94, sd=0.005. **(b)** Expected ERCC molecules per GEM vs. observed UMI counts at ERCC2 dilution of 1:50. **(c)** Conversion efficiency of each ERCC molecule as a function of their transcript GC content. **(d)** Conversion efficiency of each ERCC molecule as a function of their transcript length. **(e)** Conversion efficiency estimated from ddPCR assay of 8 genes. **(f)** CV^2 vs. mean UMI counts, where CV is the coefficient of variation, defined as the ratio of the standard deviation to the mean (on a log-log scale). The dashed line represents CV^2=1/mean.

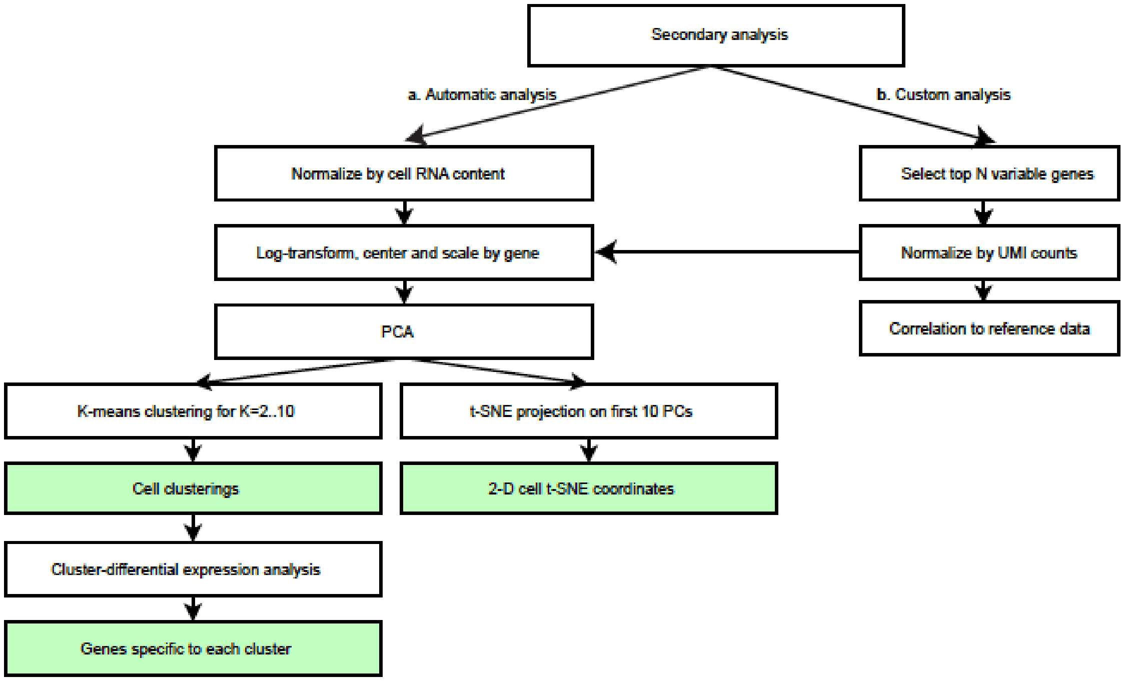

Supplementary Figure 3. Secondary analysis performed by the Cell Ranger pipeline (a), and custom analysis workflow (b).

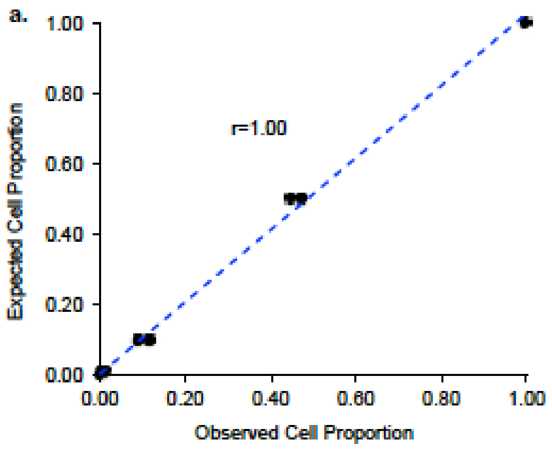

Observed Cell Proportion	Expected Cell Proportion	Number of Cells Recovered
0	0	3250
0.01	0.01	4190
0.01	0.01	4178
0.01	0.01	2676
0.09	0.1	4087
0.09	0.1	4080
0.12	0.1	3417
0.12	0.1	3419
0.45	0.5	3315
0.47	0.5	3381
0.47	0.5	3379
1	1	2901

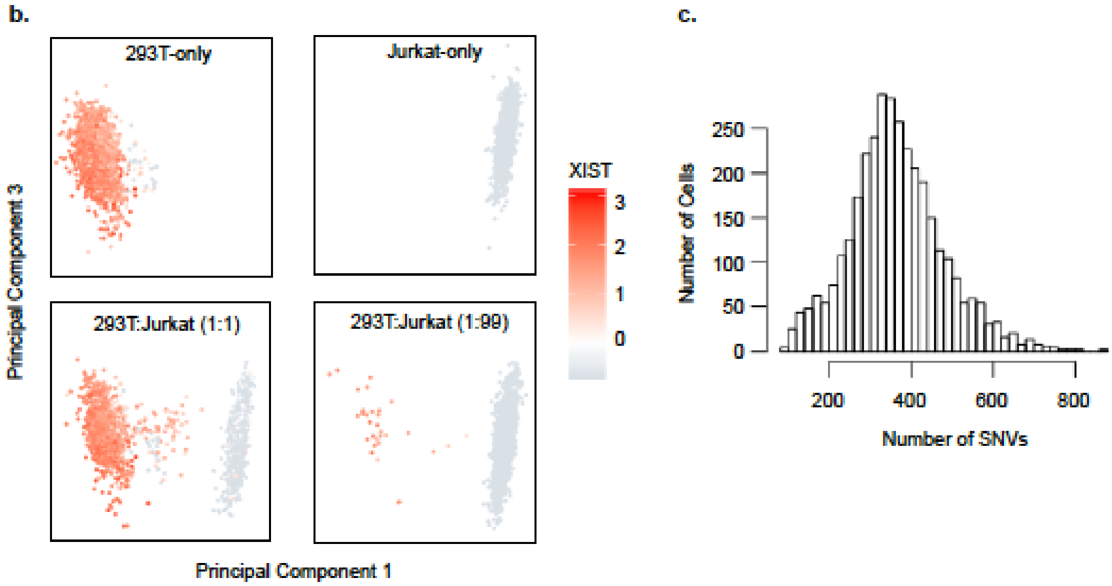

Supplementary Figure 4. Expected proportions of Jurkat and 293T cells can be detected in Jurkat:293T cell mixture. (a) Expected cell proportion is well correlated with observed cell proportion among 12 independent experiments. **(b)** Principal component 1 vs. 3 of normalized scRNA-seq data, with each cell colored by normalized expression of *XIST*. **(c)** Distribution of filtered SNVs/cell detected in 293Ts.

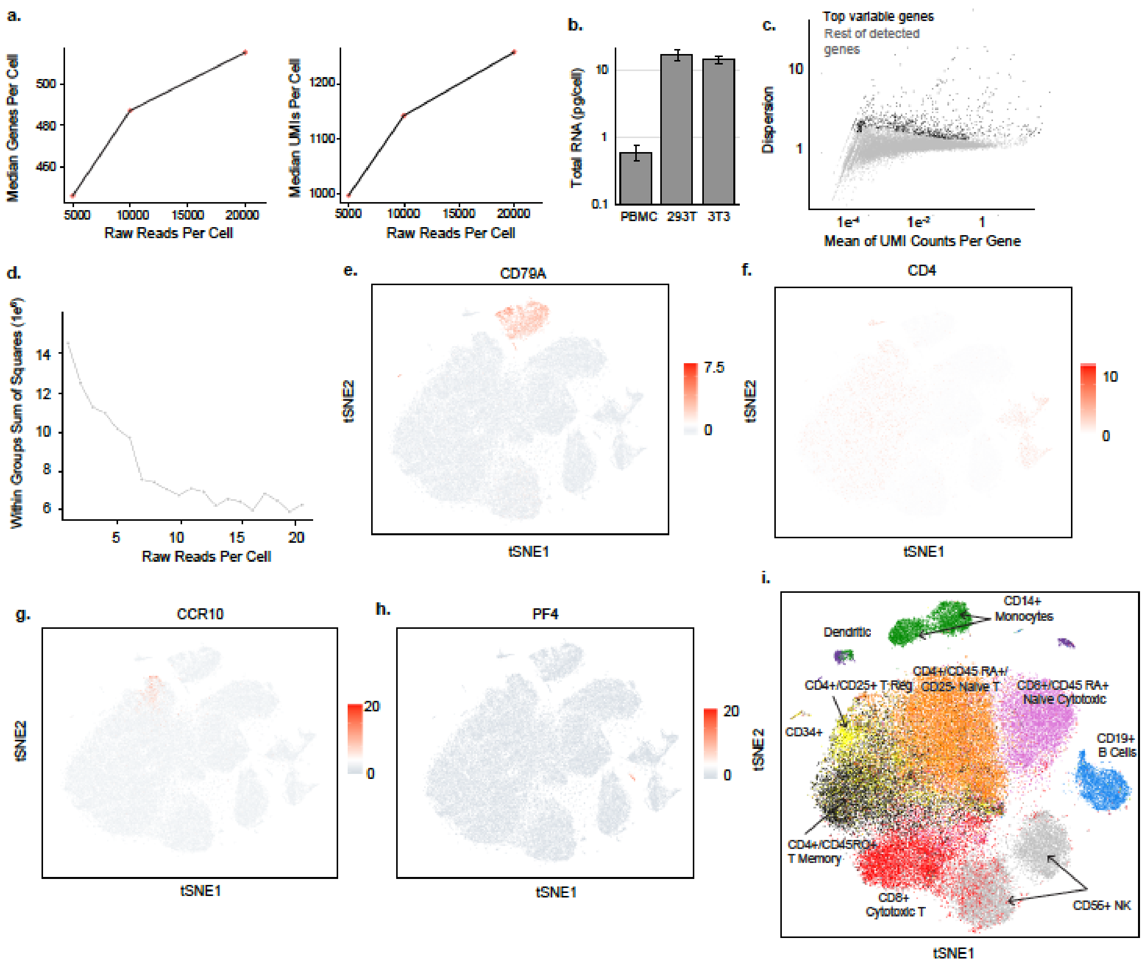

Supplementary Figure 5. Conversion efficiency and expression of marker genes in fresh PBMCs. (a) Median number of genes (left) and UMI counts (right) detected per cell as a function of raw reads per cell. **(b)** Total RNA (pg/cell) in PBMCs, 293Ts and 3T3s. (n=7 for PBMC, n=4 for 293T, n=4 for 3T3 cells, mean ± s.e.m.). **(c)** Normalized dispersion vs. mean UMI counts. Black dots represent top most variable genes used for PCA. **(d)** Within groups sum of squares vs. number of clusters for k-means clustering. **(e-h)** tSNE projection of 68k PBMCs, colored by normalized expression of *CD79A, CD4, CCR10* and *PF4* in each cell, respectively. UMI normalization was performed by first dividing UMI counts by the total UMI counts in each cell, followed by multiplication with the median of the total UMI counts across cells. Then we took the natural log of the UMI counts. Finally, each gene was normalized such that the mean signal for each gene is 0, and standard deviation is 1. **(i)** Seurat's tSNE projection of 68k PBMCs, colored by the inferred cell type assignment from purified PBMCs.

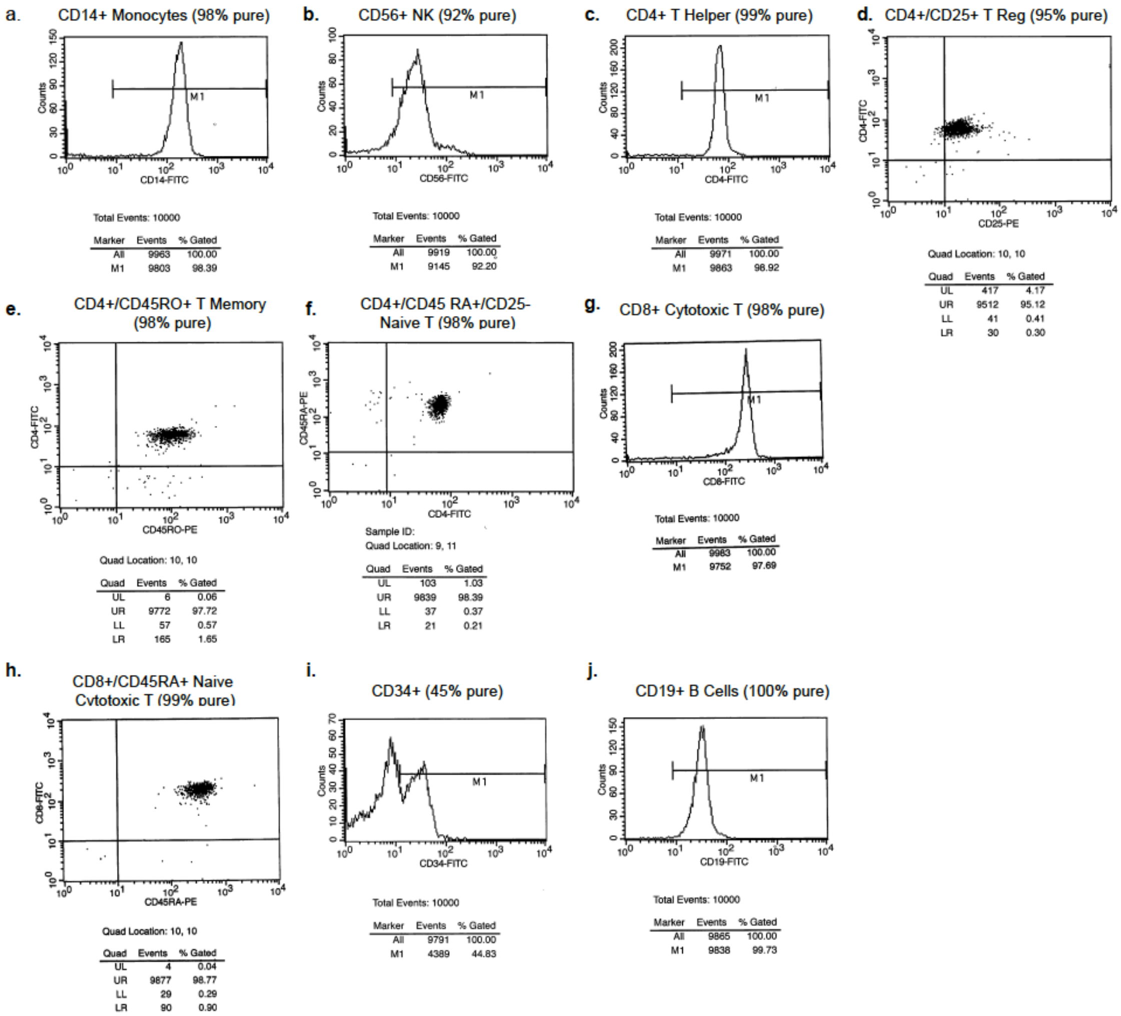

Supplementary Figure 6. FACS analysis of bead enriched sub-populations of PBMCs.

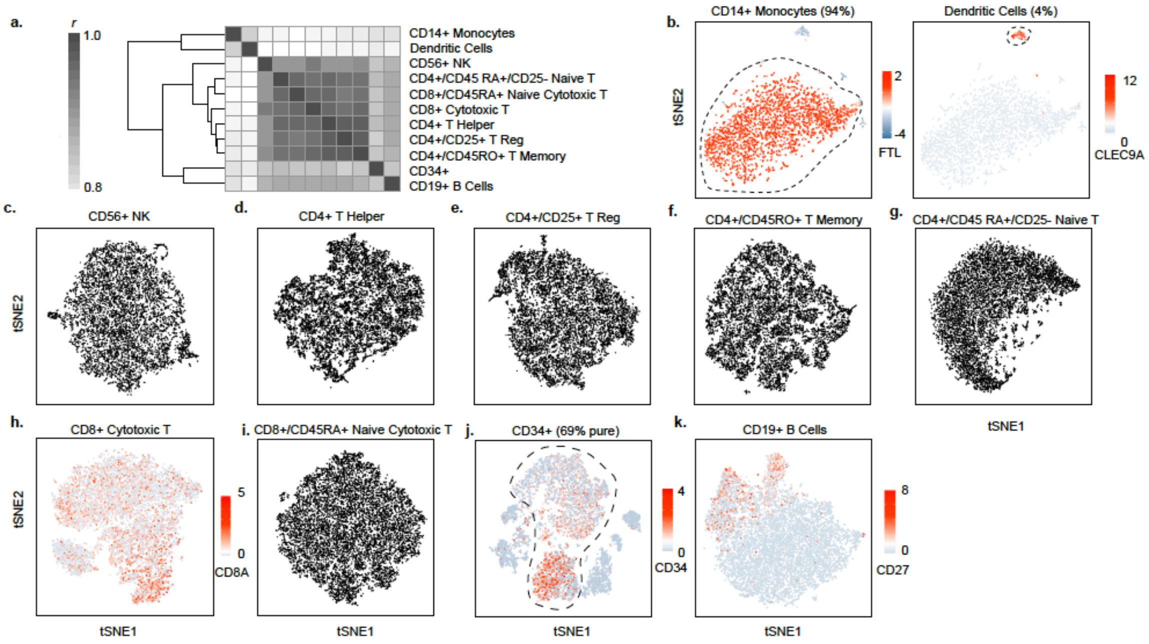

Supplementary Figure 7. tSNE projection of bead enriched sub-populations of PBMCs. (a) 11 purified sub-populations of PBMCs were used. Correlation was calculated using their average expression profile and grouped by hierarchical clustering. The heatmap displays the correlation coefficient in the pairwise comparison of sub-populations. **(b-k)** tSNE projection of each purified population. In **b, h, j, k**, each cell is colored by normalized expression of marker genes *FTL, CLEC9A, CD8A, CD34* and *CD27* respectively. UMI normalization was performed by first dividing UMI counts by the total UMI counts in each cell, followed by multiplication with the median of the total UMI counts across cells. Then we took the natural log of the UMI counts. Finally, each gene was normalized such that the mean signal for each gene is 0, and standard deviation is 1. When more than 1 population was detected in a sample (**b** and **j**), only the population showing the correct marker expression was selected (marked by a dotted polygon).

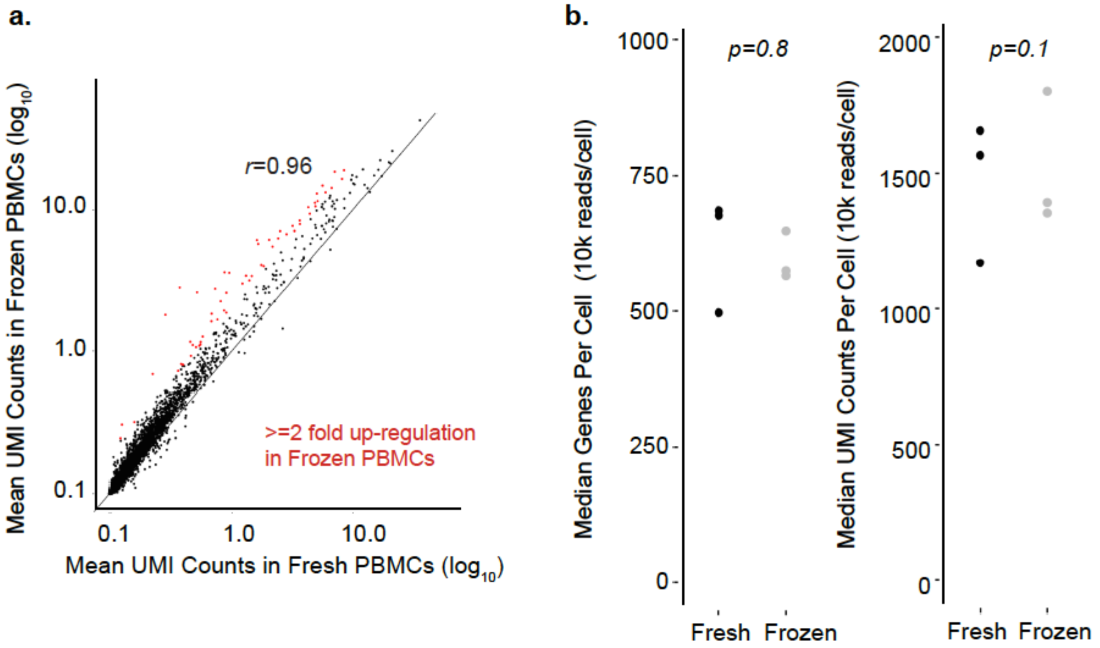

c.

PBMCs	Number of Recovered Cells	CD34+ (%)	Dendritic Cells (%)	Monocytes (%)	B (%)	T+NK (%)
Fresh	4632	0.4	1.3	4.2	9.5	84.6
	17252	1.0	2.9	11.6	7.4	77.1
	68547	0.3	1.0	5.6	5.7	87.4
Frozen	2691	0.2	2.0	8.4	7.4	82.0
	5403	0.3	6.0	13.6	5.1	75.0
	5952	0.3	6.3	13.1	5.6	74.7

Supplementary Figure 8. Comparison between fresh vs. frozen PBMCs from Donor A. (a) Scatterplot of mean UMI counts per gene across all cells between fresh vs. matched frozen PBMCs. Red dots represent genes that show 2-fold upregulation in frozen PBMCs. **(b)** Median genes (left) and UMI counts (right) detected per cell between fresh and frozen PBMCs (n=3). Black points correspond to fresh PBMCs, whereas grey points correspond to frozen PBMCs. Wilcoxon ranksum test was used to test whether the number of genes and UMI counts from fresh and frozen PBMCs were significantly different. **(c)** Proportion of major cell types detected in fresh and frozen PBMCs (n=3).

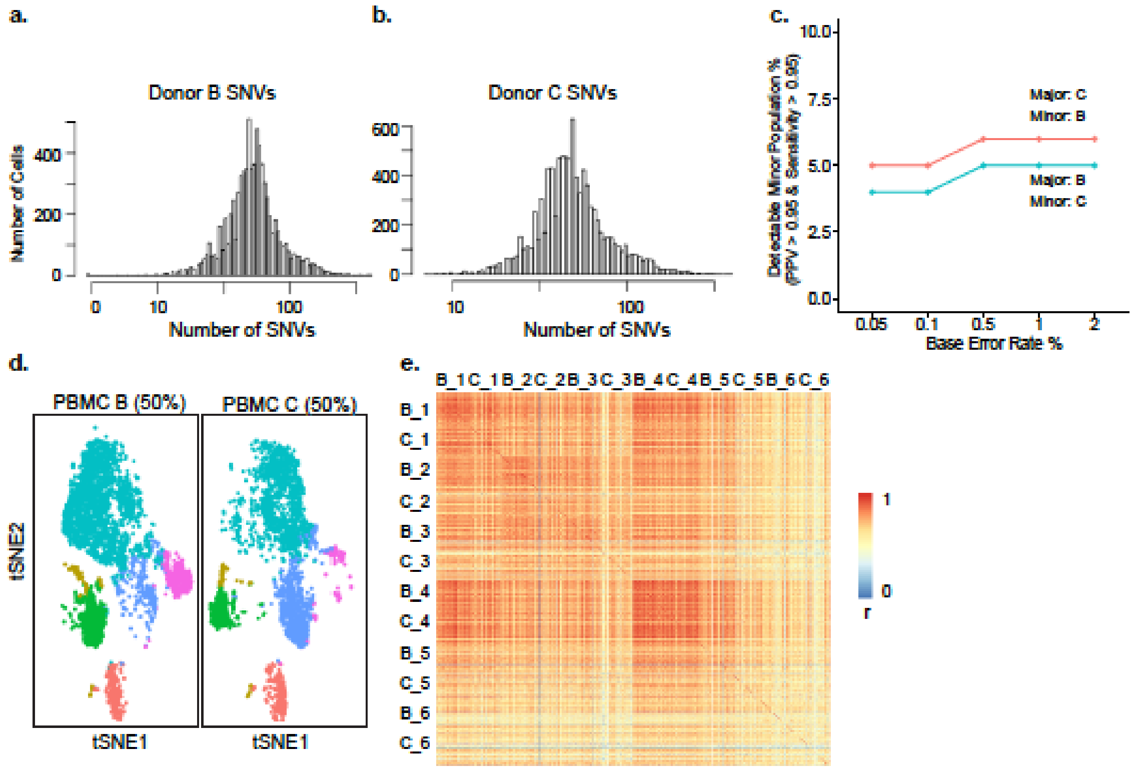

Supplementary Figure 9. SNV analysis of scRNA-seq data from Donor B and Donor C PBMCs. (a) Distribution of filtered SNVs in each PBMC from donor B. **(b)** Distribution of filtered SNVs in each PBMC from donor C. **(c)** % minor populations that can be confidently detected (PPV and sensitivity >0.95) vs. base error rate. **(d)** tSNE projection of PBMCs from Donor B and Donor C in 50:50 PBMC B:C sample, where each cell is colored based on their clustering (k-means) assignment. **(e)** Expression comparison between 5 clusters of PBMCs from donors B and C, with red indicating high similarity and blue indicating lower similarity. 100 cells were sampled from each cluster of PBMCs from donors B and C, and their pairwise gene expression was compared against each other.

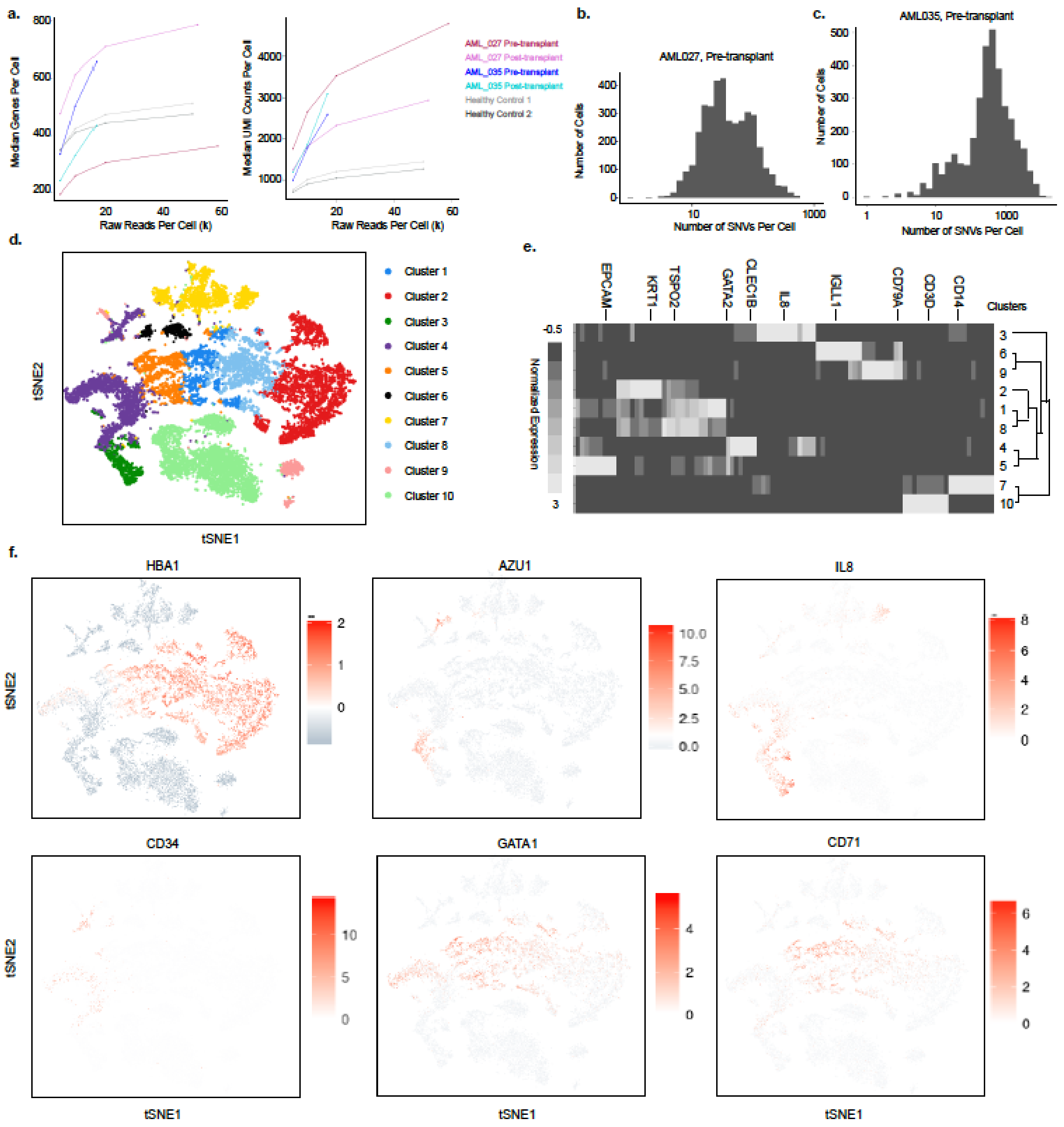

Supplementary Figure 10. Expression and clustering analyses of transplant samples. (a) Median number of genes (left) and UMIs (right) detected per cell for pre-transplant, post-transplant and BMMCs from 2 healthy donors. **(b)** Distribution of filtered SNV counts per cell in AML027 pre-transplant sample. **(c)** Distribution of filtered SNV counts per cell in AML035 pre-transplant sample. **(d)** tSNE projection of pooled 6 samples (2 healthy donors, 2 AML027 host and 2 AML035), colored by k-means clustering assignment. **(e)** Normalized expression (centered) of the top variable genes (rows) from each of 9 clusters (columns) is shown in a heatmap. Numbers on the right side indicate cluster number in **d**, with connecting lines indicating the hierarchical

relationship between clusters. Representative markers from each cluster are shown on the top. **(f)** tSNE projection of all cells, with each cell colored by normalized expression of *HBA1, AZU1, IL8, CD34, GATA1* and *CD71* respectively. UMI normalization was performed by first dividing UMI counts by the total UMI counts in each cell, followed by multiplication with the median of the total UMI counts across cells. Then we took the natural log of the UMI counts. Finally, each gene was normalized such that the mean signal for each gene is 0, and standard deviation is 1.

Supplementary Tables

Supplementary Table 1. Sequencing metrics summary of all the scRNA-seq data.

Description	Sequencer	UMI Length	Number of Cells Recovered	Mean Reads per Cell*	Median Genes per Cell*	Median UMI Counts per Cell*	Fraction reads usable	Fraction Reads in Cells	Reads Mapped Confidently to Exonic Regions	Reads Mapped Confidently to Intronic Regions	Reads Mapped Confidently to Intergenic Regions	Fraction reads unmapped	Valid Barcodes	Valid UMIs	Fraction corrected barcodes	Fraction corrected UMIs	Median insert size	cDNA PCR Duplication*
293T and 3T3 Cell Mixture	NextSeq 500 High Output	10	1,012	106,237	4,738 and 4,233	25,070 and 27,198	56.50%	83.9% and 84.5%	38.2% and 33.2%	3.8% and 1.9%	1.2% and 1.3%	5.00%	94.80%	99.30%	6.90%	2.70%	222 and 225	50.10%
293T Cells	HiSeq 2500 Rapid Run V2	10	2,885	33,406	3,393	14,318	62.20%	90.00%	76.30%	8.00%	2.90%	2.50%	95.10%	99.50%	13.00%	0.50%	211	26.00%
Jurkat Cells	HiSeq 2500 Rapid Run V2	10	3,258	33,361	3,204	14,604	64.70%	93.70%	75.50%	8.40%	3.10%	3.00%	96.40%	99.50%	8.20%	0.40%	223	28.40%
50%:50% Jurkat:293T Cell Mixture	NextSeq 500 High Output	10	3,388	33,354	3,408	13,987	62.50%	92.30%	75.20%	9.10%	2.70%	3.80%	94.50%	99.40%	7.30%	2.40%	207	27.40%
99%:1% Jurkat:293T Cell Mixture	NextSeq 500 High Output	10	4,185	32,945	3,366	13,633	63.40%	92.50%	75.90%	8.30%	2.80%	4.10%	94.60%	99.40%	7.20%	2.60%	204	29.90%
ERCC (1k GEMS, 1:10 Dilution)	HiSeq 2500 Rapid Run V2	10	1,015	247,934	49	11,125	91.90%	94.00%	99.20%	0.00%	0.00%	0.70%	98.30%	99.50%	2.70%	3.30%	256	95.30%
Fresh 68k PBMCs (Donor A)	NextSeq 500 High Output	5	68,579	20,491	525	1,292	68.40%	95.60%	77.40%	8.20%	2.50%	2.20%	95.20%	98.40%	5.50%	6.40%	242	88.30%
CD34+ Cells	NextSeq 500 High Output	5	9,232	24,735	1,274	4,168	65.70%	93.30%	76.20%	8.00%	2.40%	2.00%	96.10%	98.40%	4.40%	11.50%	238	73.50%
CD56+ Natural Killer Cells	NextSeq 500 High Output	5	8,385	29,288	710	1,580	65.20%	91.70%	77.40%	9.90%	2.90%	2.00%	96.30%	97.60%	3.70%	6.00%	248	90.80%
CD4+/CD45RA+/CD25- Naive T cells	NextSeq 500 High Output	5	10,479	19,405	480	1,186	70.50%	95.90%	79.10%	5.70%	2.20%	1.90%	96.70%	98.40%	3.40%	4.90%	249	90.70%
CD4+/CD25+ Regulatory T Cells	NextSeq 500 High Output	5	10,263	26,935	547	1,223	65.30%	90.00%	78.30%	6.60%	2.50%	2.20%	96.60%	98.30%	3.50%	4.40%	245	92.30%
CD8+/CD45RA+ Naive Cytotoxic T Cells	NextSeq 500 High Output	5	11,953	19,999	502	1,442	71.30%	97.50%	78.80%	5.40%	2.10%	2.10%	96.70%	98.30%	3.30%	6.00%	243	88.10%
CD4+/CD45RO+ Memory T Cells	NextSeq 500 High Output	5	10,224	24,441	557	1,506	70.70%	98.50%	77.90%	7.40%	2.20%	2.20%	96.50%	98.20%	3.60%	5.90%	251	90.40%
CD8+ Cytotoxic T cells	NextSeq 500 High Output	5	10,209	28,618	573	1,630	69.10%	95.30%	78.20%	6.30%	2.10%	2.00%	96.50%	98.30%	3.90%	6.90%	240	91.20%
CD19+ B Cells	NextSeq 500 High Output	5	10,085	25,286	478	1,237	64.40%	92.80%	74.80%	9.40%	3.50%	2.10%	96.50%	98.40%	4.00%	5.30%	249	90.80%
CD4+ T Helper Cells	NextSeq 500 High Output	5	11,213	21,329	546	1,326	70.20%	95.20%	79.40%	5.50%	2.20%	2.10%	96.60%	98.40%	3.50%	5.30%	248	90.40%
CD14+ Monocytes	NextSeq 500 High Output	5	2,612	102,430	382	782	37.70%	53.60%	76.40%	9.10%	3.50%	3.20%	96.40%	98.40%	3.70%	3.90%	237	96.70%
Frozen PBMCs (Donor A)	NextSeq 500 High Output	5	2,900	24,722	722	2,117	66.10%	95.90%	75.10%	8.20%	2.70%	3.00%	94.30%	98.10%	6.40%	8.00%	216	85.70%
Frozen PBMCs (Donor B)	HiSeq 2500 Rapid Run V2	10	7,783	14,082	652	1,653	62.20%	96.60%	70.30%	15.60%	3.20%	1.80%	96.20%	99.70%	9.70%	0.60%	204	78.60%
Frozen PBMCs (Donor C)	HiSeq 2500 Rapid Run V2	10	9,519	13,999	671	1,804	62.30%	97.20%	70.40%	14.90%	3.30%	1.60%	96.40%	99.30%	9.60%	0.60%	211	76.90%
50%:50% Donor B: Donor C PBMC Mixture	HiSeq 2500 Rapid Run V2	10	8,186	14,147	654	1,650	62.00%	96.40%	70.10%	14.90%	3.50%	1.80%	96.20%	99.30%	9.60%	0.60%	206	78.70%
90%:10% Donor B: Donor C PBMC Mixture	HiSeq 2500 Rapid Run V2	10	7,046	14,128	621	1,522	61.90%	96.10%	70.30%	15.50%	3.20%	1.70%	96.20%	99.30%	9.70%	0.60%	209	80.50%
99%:1% Donor B: Donor C PBMC Mixture	HiSeq 2500 Rapid Run V2	10	8,340	14,007	658	1,616	62.20%	96.60%	70.30%	15.10%	3.40%	1.80%	96.20%	99.30%	9.70%	0.60%	206	78.80%
Frozen BMMCs (Healthy Control 1)	HiSeq 2500 Rapid Run V2	5	1,985	135,048	567	1,970	54.20%	80.90%	73.30%	7.80%	3.60%	2.60%	94.30%	98.70%	7.50%	20.90%	218	95.60%
Frozen BMMCs (Healthy Control 2)	HiSeq 2500 Rapid Run V2	5	2,472	89,643	508	1,552	56.50%	87.10%	71.00%	10.10%	4.00%	2.50%	94.60%	98.70%	7.20%	17.20%	225	95.10%
AML027 Pre-transplant BMMCs	NextSeq 500 High Output	10	3,933	58,491	369	4,387	63.40%	93.40%	74.50%	3.30%	1.50%	4.60%	92.30%	99.40%	9.20%	6.10%	191	83.60%
AML027 Post-transplant BMMCs	NextSeq 500 High Output	10	3,965	51,136	785	3,010	50.20%	84.60%	68.70%	10.00%	2.90%	8.30%	92.60%	99.30%	8.40%	3.90%	212	83.90%
AML035 Pre-transplant BMMCs	NextSeq 500 High Output	10	3,592	16,593	662	2,596	42.70%	65.30%	71.70%	8.50%	1.90%	4.70%	92.80%	99.30%	8.90%	3.80%	209	35.00%
AML035 Post-transplant BMMCs	NextSeq 500 High Output	10	908	40,927	439	3,179	51.70%	88.10%	64.00%	5.60%	2.20%	13.30%	92.20%	99.30%	8.90%	3.70%	189	77.30%

* Sequencing depth dependent

Supplementary Table 2. Cell capture rate from 4 cell lines, and 17 independent samples.

Cell Types	Number of Cells Loaded	Number of Cells Recovered	Cell Capture Rate
HCC38	2,304	1,499	65%
HCC38	5,760	3,067	53%
HCC38	17,280	9,354	54%
HCC38	23,040	12,057	52%
3T3	1,152	535	46%
3T3	2,304	1,177	51%
3T3	4,032	1,942	48%
3T3	5,760	2,745	48%
293T	1,152	483	42%
293T	2,304	1,033	45%
293T	4,032	1,769	44%
293T	5,760	2,539	44%
PBMC	2,304	1,001	43%
PBMC	5,760	2,691	47%
PBMC	11,520	5,952	52%
PBMC	17,280	7,467	43%
PBMC	23,040	10,123	44%

Supplementary Table 3. Total number of filtered SNVs and median number of filtered SNV/cell.

Samples	Total # of Filtered SNVs detected	Median # of Filtered SNVs detected per cell
293T Cells	19,595	321
Jurkat Cells	22,171	387
50%:50% Jurkat:293T Cell Mixture	26,108	368
99%:1% Jurkat:293T Cell Mixture	27,950	416
Frozen PBMCs From Donor B	14,157	55
Frozen PBMCs From Donor C	16,293	49
50%:50% Donor B: Donor C PBMC Mixture	14,868	47
90%:10% Donor B: Donor C PBMC Mixture	12,348	49
99%:1% Donor B: Donor C PBMC Mixture	14,165	55
AML027 Pre-transplant BMMCs	8,900	37
AML027 Post-transplant BMMCs	12,374	80
AML035 Pre-transplant BMMCs	9,342	61
AML035 Post-transplant BMMCs	4,510	37

Supplementary Table 4. Bead-purification strategy of bead enriched PBMCs from Donor A.

Cell types	Catalog numbers	Isolation methods
CD34+ cells	C-PB116-0.2M	Isolation kit from Milteny 130-046-701
CD14+ Monocytes	C-PB114-10M7	Negative selection using Stemcell 19059
CD19+ B cells	C-PB106-10M7	Negative selection from Stemcell 19054
CD56+ NK cells	C-PB118-5M6	Negative selection from Stemcell 19055
CD8+ Cytotoxic T cells	C-PB105-10M	Negative selection from Stemcell 19053
CD8+/CD45RA+ Naīve Cytotoxic T cells	C-PB125-5M3	Negative selection from Stemcell 19058
CD4+/CD45RO+ Memory T cells	C-PB124-5M3	Negative selection from Stemcell 19157
CD4+/CD45RA+/CD25- Naīve T cells	C-PB123-5M	Negative selection from Stemcell 19155
CD4+/CD25+ Regulatory T cells	C-PB122-2M4	Isolation kit from Stemcell 19052 to isolate CD4, then isolate CD25 with Miltenyi 130-092-983
CD4+ Helper T	C-PB103-20M	Negative selection using Stemcell 19052

Supplementary Table 5. List of genes that show 2-fold upregulation in scRNA-seq data of frozen PBMCs from Donor A.

Gene ID	Mean UMI Counts (Frozen PBMCs)	Mean UMI Counts (Fresh PBMCs)	Log2 Fold Change (Frozen vs. Fresh)
S100A11	1.16	0.45	1.36
S100A9	2.82	0.37	2.92
S100A8	1.81	0.28	2.67
S100A6	3.14	1.39	1.17
RPS27	14.23	6.65	1.10
FCER1G	1.10	0.48	1.21
OST4	1.11	0.55	1.01
RPL31	11.45	5.12	1.16
RPL37A	6.08	1.61	1.91
RPL35A	9.36	4.41	1.08
RPL37	5.72	1.65	1.79
RPS23	10.55	4.90	1.10
COX7C	1.63	0.68	1.26
CD14	0.31	0.12	1.31
LST1	0.93	0.46	1.01
AIF1	1.16	0.55	1.07
RPS10	3.40	1.31	1.38
RPS12	18.94	8.43	1.17
TOMM7	2.25	0.81	1.48
TMEM176B	0.32	0.16	1.04
RPL36A	2.59	0.90	1.52
RPS20	7.06	3.29	1.10
RPL30	10.40	4.28	1.28
RPL35	8.38	3.64	1.20
FCN1	0.69	0.22	1.63
RPS24	6.26	2.42	1.37
RPLP2	18.52	7.07	1.39
MS4A6A	0.25	0.12	1.03
FAU	7.90	3.65	1.11
C12orf57	0.81	0.38	1.09
RPS26	4.06	1.75	1.21
LYZ	2.61	0.52	2.33
TPT1	12.96	5.05	1.36
RPS29	2.76	0.73	1.92
RPLP1	16.44	8.12	1.02
TCEB2	0.80	0.40	1.02
RPS15A	13.23	5.94	1.16
RPL23	3.00	1.23	1.29
RPL27	7.04	2.51	1.49
RPL38	3.57	0.96	1.90
ZFAS1	1.06	0.51	1.07
ATP5E	1.94	0.86	1.17
RPS21	3.60	0.87	2.05
RPL36	6.69	2.82	1.25
RPS28	6.10	2.04	1.58
UBL5	0.73	0.36	1.01
UBA52	7.67	3.18	1.27
COX6B1	1.09	0.54	1.01
HCST	1.67	0.76	1.14
TYROBP	1.84	0.68	1.44
RPS16	11.15	4.87	1.20
RPS11	5.46	2.17	1.33
RPL28	14.78	5.61	1.40
LGALS1	1.27	0.58	1.14
RP11-763B22.6	3.98	1.83	1.12
RP11-403I13.5	3.39	1.46	1.21
FCGR1C	1.87	0.90	1.06

Supplementary Table 6. Comparison between GemCode single cell technology and representative single cell RNA-seq approaches.

Supplementary References

1. Jaitin, D.A. *et al.* Massively parallel single-cell RNA-seq for marker-free decomposition of tissues into cell types. *Science* 343, 776-779 (2014).

2. Pollen, A.A. *et al.* Low-coverage single-cell mRNA sequencing reveals cellular heterogeneity and activated signaling pathways in developing cerebral cortex. *Nat. Biotechnol.* 32, 1053-1058 (2014).

3. Fluidigm, Single-Cell Whole Genome Sequencing on the C1 System: a Performance Evaluation https://www.fluidigm.com/binaries/content/documents/fluidigm/marketing/single-cell-whole-genome-sequencing/single-cell-whole-genome-sequencing/fluidigm%3Afile (2016).

4. Macosko, E.Z. *et al.* Highly Parallel Genome-wide Expression Profiling of Individual Cells Using Nanoliter Droplets. *Cell* 161, 1202-1214 (2015).

5. Klein, A.M. *et al.* Droplet barcoding for single-cell transcriptomics applied to embryonic stem cells. *Cell* 161, 1187-1201 (2015).

6. Soumillon, M., Cacchiarelli, D., Semrau, S., van Oudenaarden, A. & Mikkelsen, T.S. Characterization of directed differentiation by high-throughput single-cell RNA-Seq. *bioRxiv* (2016).
